# Supplementary material for: Cis-regulatory basis of sister cell type divergence in the vertebrate retina
Source: eLife. 2019 Oct 21;8:e48216. doi: 10.7554/eLife.48216 (PMC6802965; doi:10.7554/eLife.48216)
Supplement: Figure 2—source data 1. — Dot plots present single cell expression data obtained by Shekhar et al. (2016) using Drop-seq for genes identified in this study by bulk RNA-seq as differentially enriched in ON or OFF bipolar cells. Drop-seq data was available for 630 of the 680 genes, which are sorted by lowest adjusted p-value. (A-H) Genes enriched in OFF bipolar cells. (I-O) Genes enriched in ON bipolar cells. nTrans = mean number of transcripts expressed per cell in each cluster identified as a bipolar cell type. PercExpr = percentage of cells within each cluster found to express the indicated gene. [file elife-48216-fig2-data1.pdf]

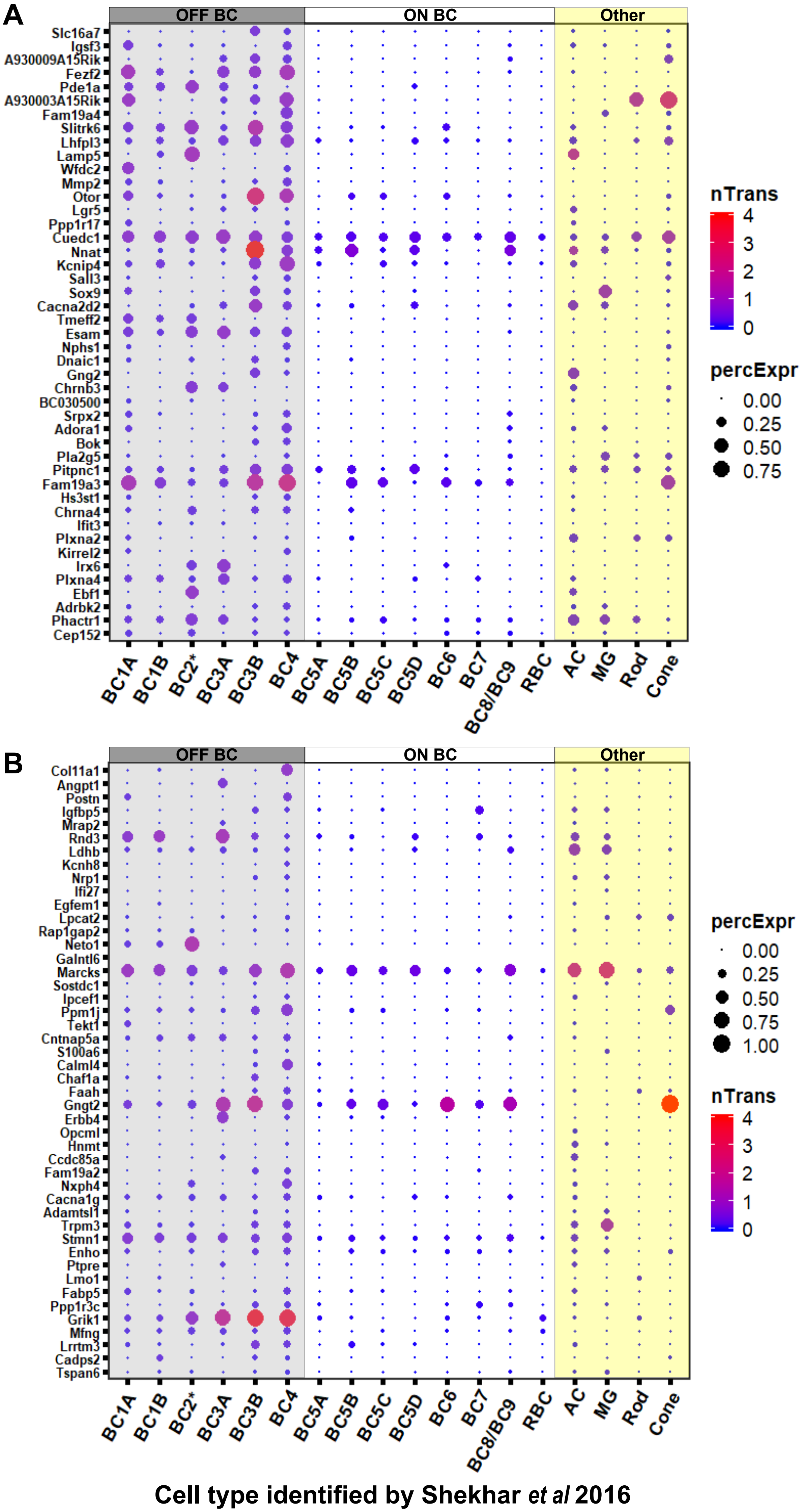
A
OFF BC
ON BC
Other
Slc16a7
Igsf3
A930009A15Rik
Fezf2
Pde1a
A930003A15Rik
Fam19a4
Slitrk6
Lhfpl3
Lamp5
Wfdc2
Mmp2
Otor
Lgr5
Ppp1r17
Cuedc1
Nnat
Kcnip4
Sall3
Sox9
Cacna2d2
Tmeff2
Esam
Nphs1
Dnaic1
Gng2
Chrnb3
BC030500
Srpx2
Adora1
Bok
Pla2g5
Pitpnc1
Fam19a3
Hs3st1
Chrna4
Ifit3
Plxna2
Kirrel2
Irx6
Plxna4
Ebf1
Adrbk2
Phactr1
Cep152
nTrans
4
3
2
1
0
percExpr
0.00
0.25
0.50
0.75
BC1A
BC1B
BC2*
BC3A
BC3B
BC4
BC5A
BC5B
BC5C
BC5D
BC6
BC7
BC8/BC9
RBC
AC
MG
Rod
Cone
B
OFF BC
ON BC
Other
Col11a1
Angpt1
Postn
Igfbp5
Mrap2
Rnd3
Ldhb
Kcnh8
Nrp1
Ifi27
Egfem1
Lpcat2
Rap1gap2
Neto1
Galntl6
Marcks
Sostdc1
Ipcef1
Ppm1j
Tekt1
Cntnap5a
S100a6
Calml4
Chaf1a
Faah
Gngt2
Erbb4
Opcml
Hnmt
Ccdc85a
Fam19a2
Nxph4
Cacna1g
Adamtsl1
Trpm3
Stmn1
Enho
Ptpre
Lmo1
Fabp5
Ppp1r3c
Grik1
Mfng
Lrrtm3
Cadps2
Tspan6
percExpr
0.00
0.25
0.50
0.75
1.00
nTrans
4
3
2
1
0
BC1A
BC1B
BC2*
BC3A
BC3B
BC4
BC5A
BC5B
BC5C
BC5D
BC6
BC7
BC8/BC9
RBC
AC
MG
Rod
Cone
Cell type identified by Shekhar et al 2016

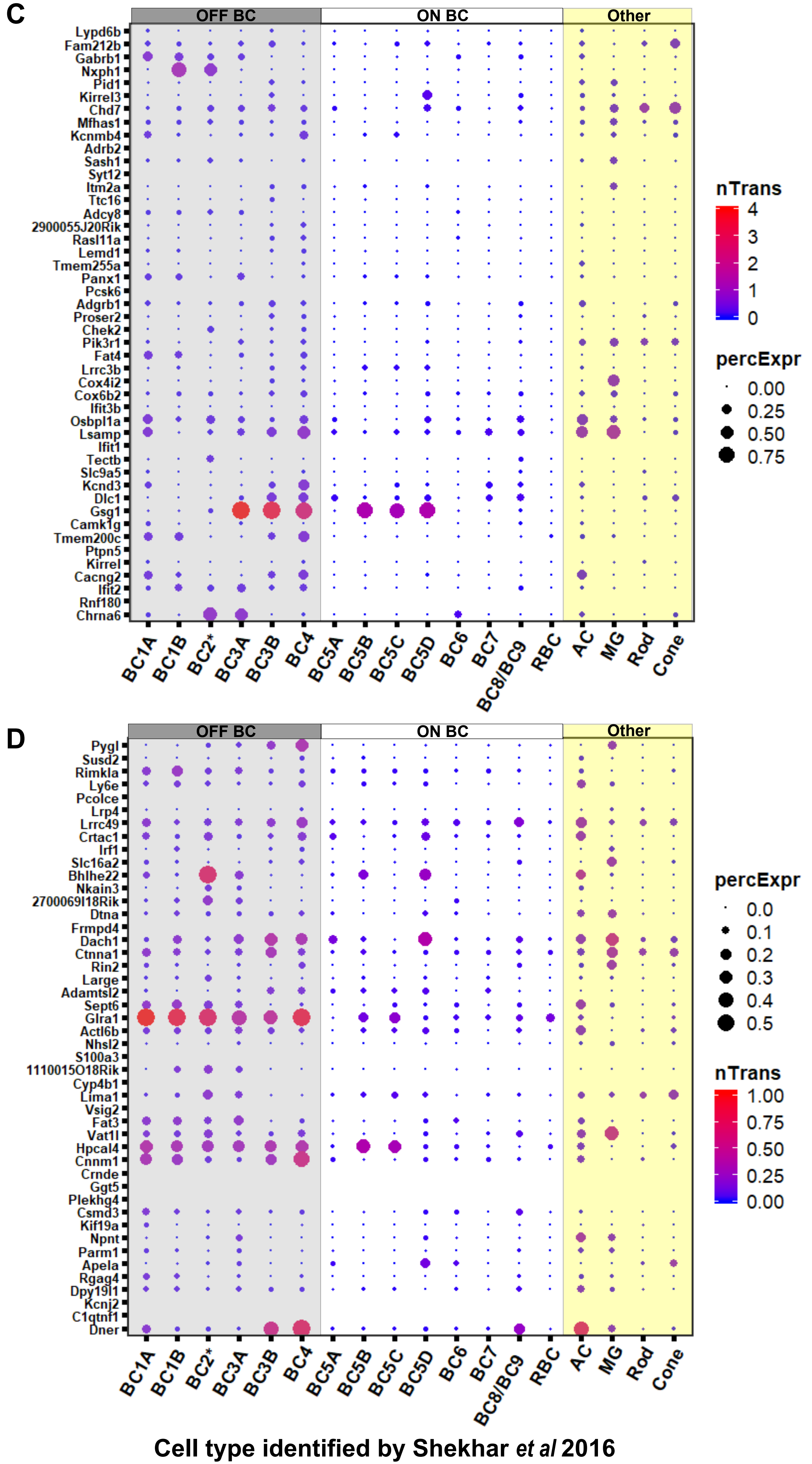
C
OFF BC
ON BC
Other
Lypd6b
Fam212b
Gabrb1
Nxph1
Pid1
Kirrel3
Chd7
Mfhas1
Kcnmb4
Adrb2
Sash1
Syt12
Itm2a
Ttc16
Adcy8
2900055J20Rik
Rasl11a
Lemd1
Tmem255a
Panx1
Pcsk6
Adgrb1
Proser2
Chek2
Pik3r1
Fat4
Lrrc3b
Cox4i2
Cox6b2
Ifit3b
Osbpl1a
Lsamp
Ifit1
Tectb
Slc9a5
Kcnd3
Dlc1
Gsg1
Camk1g
Tmem200c
Ptpn5
Kirrel
Cacng2
Ifit2
Rnf180
Chrna6
nTrans
4
3
2
1
0
percExpr
0.00
0.25
0.50
0.75
BC1A
BC1B
BC2*
BC3A
BC3B
BC4
BC5A
BC5B
BC5C
BC5D
BC6
BC7
BC8/BC9
RBC
AC
MG
Rod
Cone
D
OFF BC
ON BC
Other
Pygl
Susd2
Rimkla
Ly6e
Pcolce
Lrp4
Lrrc49
Crtac1
Irf1
Slc16a2
Bhlhe22
Nkain3
2700069I18Rik
Dtna
Frmpd4
Dach1
Ctnna1
Rin2
Large
Adamtsl2
Sept6
Glra1
Actl6b
Nhsl2
S100a3
1110015O18Rik
Cyp4b1
Lima1
Vsig2
Fat3
Vat1l
Hpcal4
Cnnm1
Crnde
Ggt5
Plekhg4
Csmd3
Kif19a
Npnt
Parm1
Apela
Rgag4
Dpy19l1
Kcnj2
C1qtnf1
Dner
percExpr
0.0
0.1
0.2
0.3
0.4
0.5
nTrans
1.00
0.75
0.50
0.25
0.00
BC1A
BC1B
BC2*
BC3A
BC3B
BC4
BC5A
BC5B
BC5C
BC5D
BC6
BC7
BC8/BC9
RBC
AC
MG
Rod
Cone
Cell type identified by Shekhar et al 2016

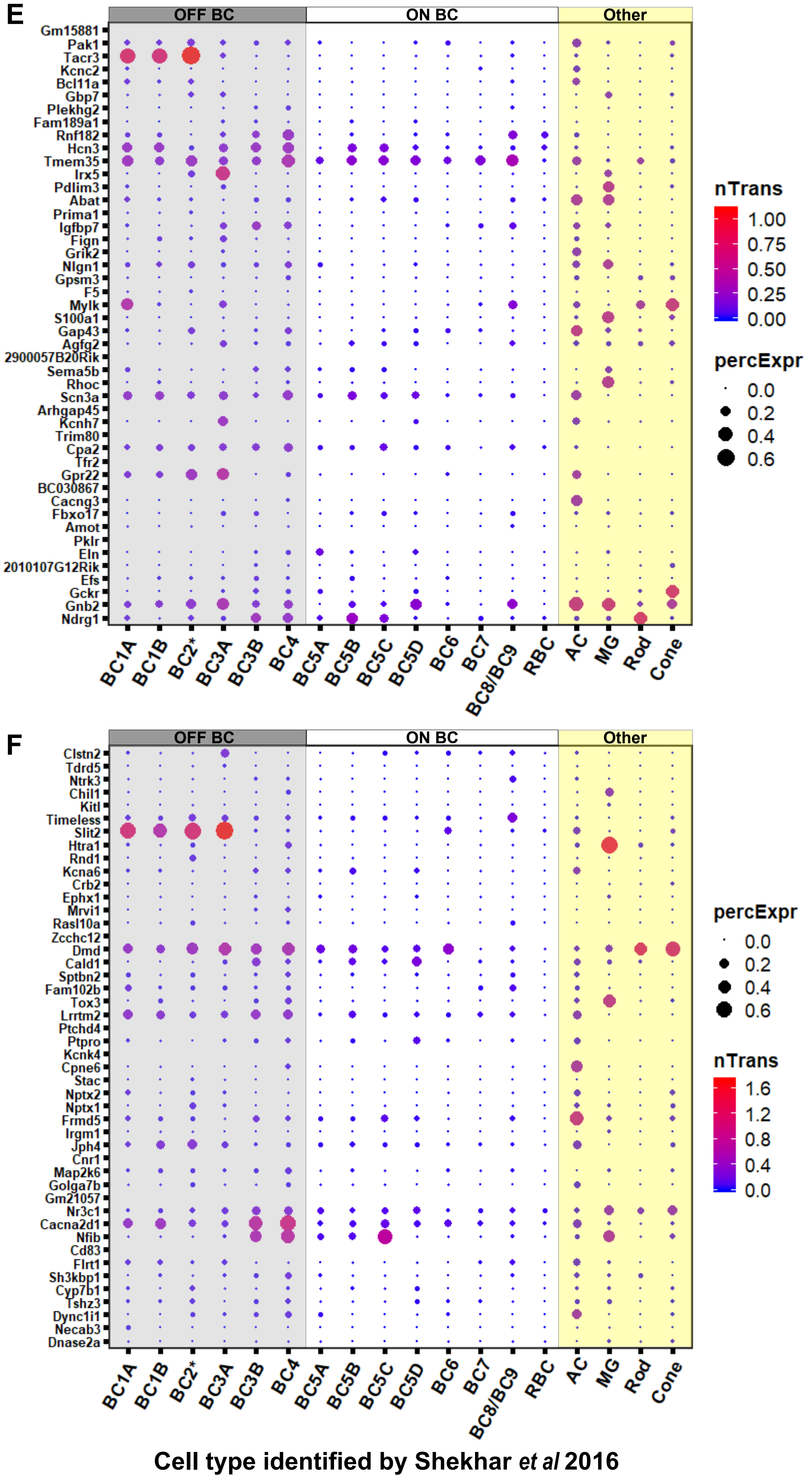

E
OFF BC
ON BC
Other
Gm15881
Pak1
Tacr3
Kcnc2
Bcl11a
Gbp7
Plekhg2
Fam189a1
Rnf182
Hcn3
Tmem35
Irx5
Pdlim3
Abat
Prima1
Igfbp7
Fign
Grik2
Nlgn1
Gpsm3
F5
Mylk
S100a1
Gap43
Agfg2
2900057B20Rik
Sema5b
Rhoc
Scn3a
Arhgap45
Kcnh7
Trim80
Cpa2
Tfr2
Gpr22
BC030867
Cacng3
Fbxo17
Amot
Pklr
Eln
2010107G12Rik
Efs
Gckr
Gnb2
Ndrg1
BC1A
BC1B
BC2*
BC3A
BC3B
BC4
BC5A
BC5B
BC5C
BC5D
BC6
BC7
BC8/BC9
RBC
AC
MG
Rod
Cone
nTrans
1.00
0.75
0.50
0.25
0.00
percExpr
0.0
0.2
0.4
0.6
F
OFF BC
ON BC
Other
Clstn2
Tdrd5
Ntrk3
Chil1
Kitl
Timeless
Slit2
Htra1
Rnd1
Kcna6
Crb2
Ephx1
Mrvi1
Rasl10a
Zcchc12
Dmd
Cald1
Sptbn2
Fam102b
Tox3
Lrrtm2
Ptchd4
Ptpro
Kcnk4
Cpne6
Stac
Nptx2
Nptx1
Frmd5
Irgm1
Jph4
Cnr1
Map2k6
Golga7b
Gm21057
Nr3c1
Cacna2d1
Nfib
Cd83
Flrt1
Sh3kbp1
Cyp7b1
Tshz3
Dync1i1
Necab3
Dnase2a
BC1A
BC1B
BC2*
BC3A
BC3B
BC4
BC5A
BC5B
BC5C
BC5D
BC6
BC7
BC8/BC9
RBC
AC
MG
Rod
Cone
percExpr
0.0
0.2
0.4
0.6
nTrans
1.6
1.2
0.8
0.4
0.0
Cell type identified by Shekhar et al 2016

G

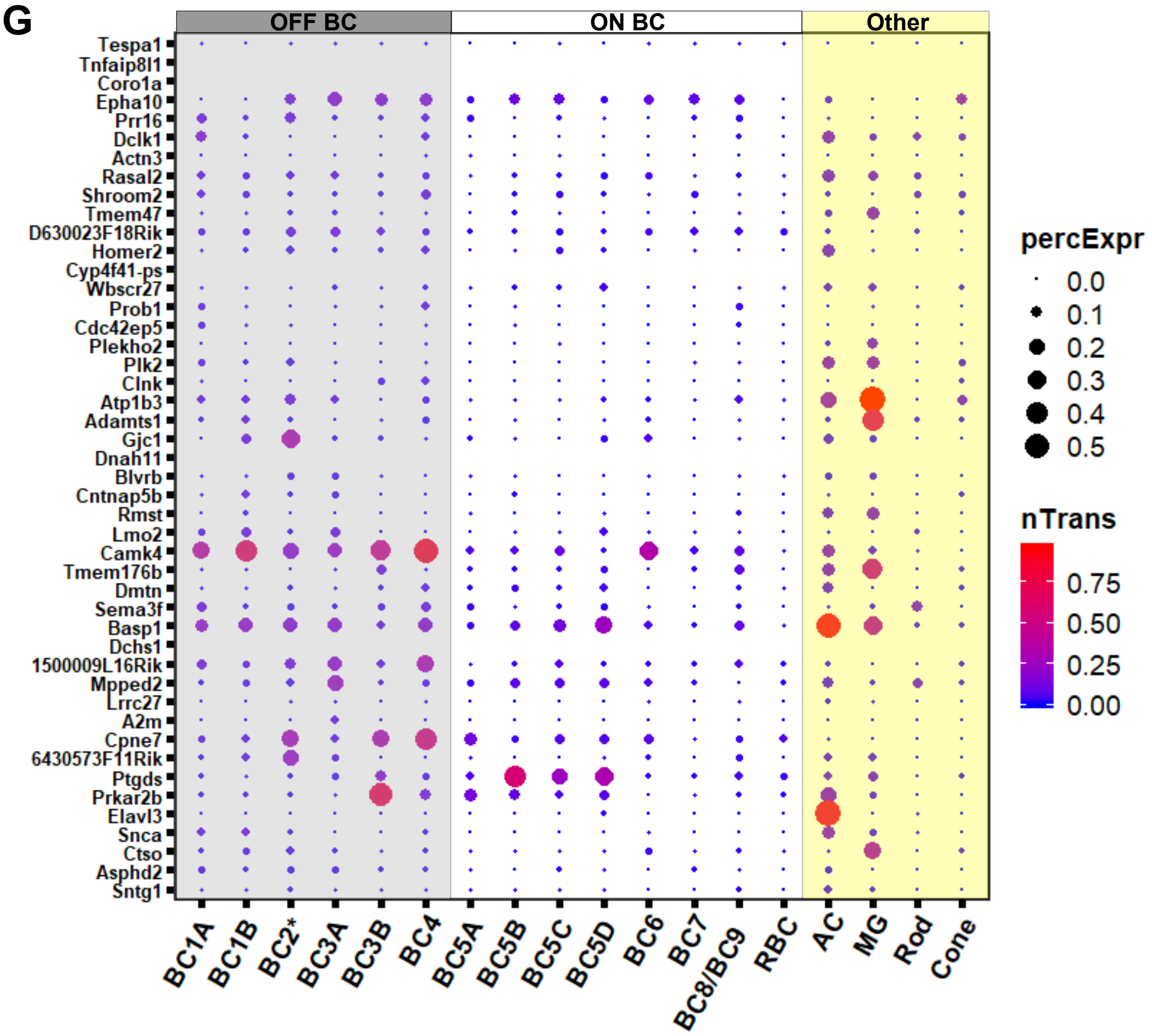

H

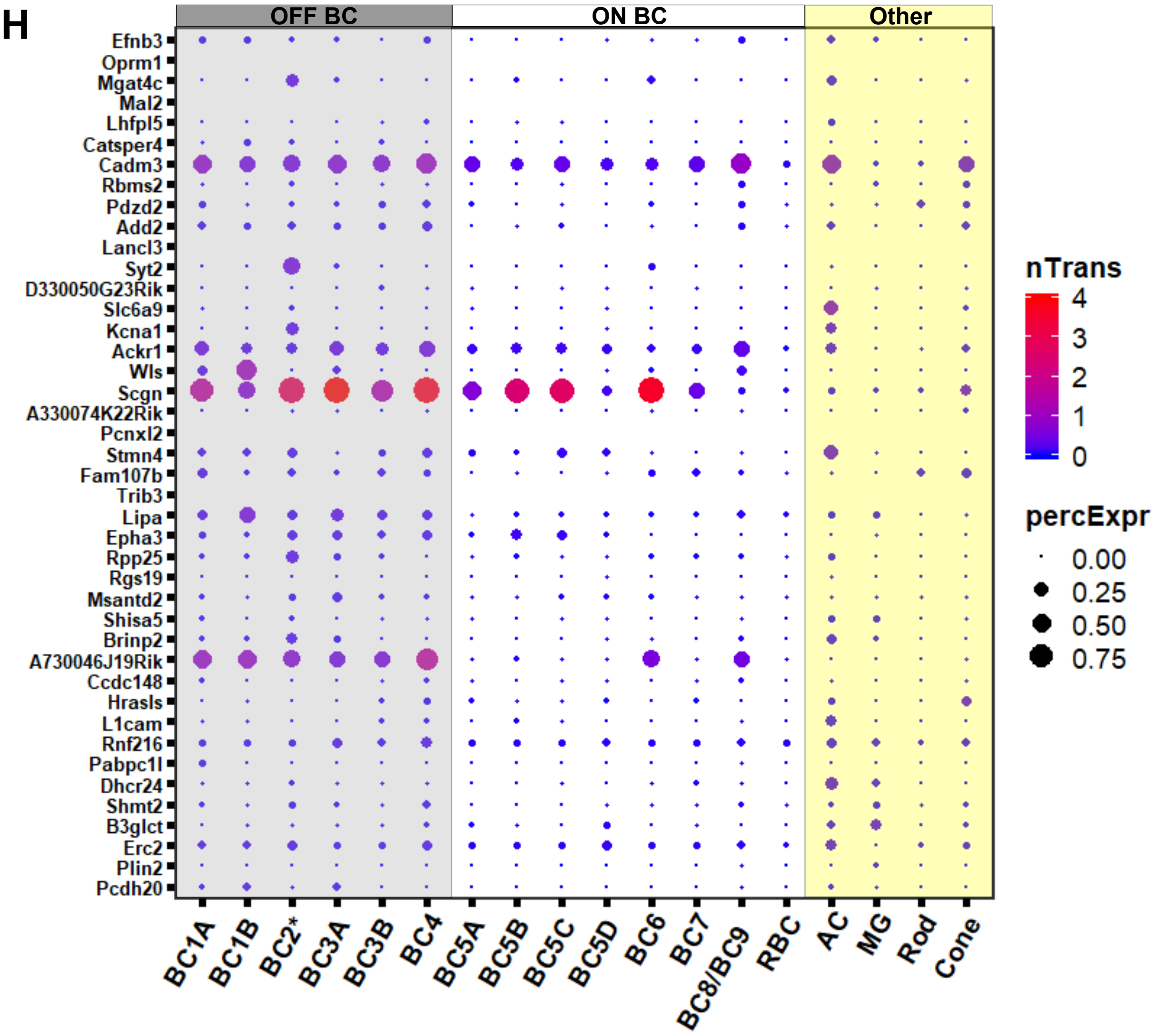

Cell type identified by Shekhar *et al* 2016

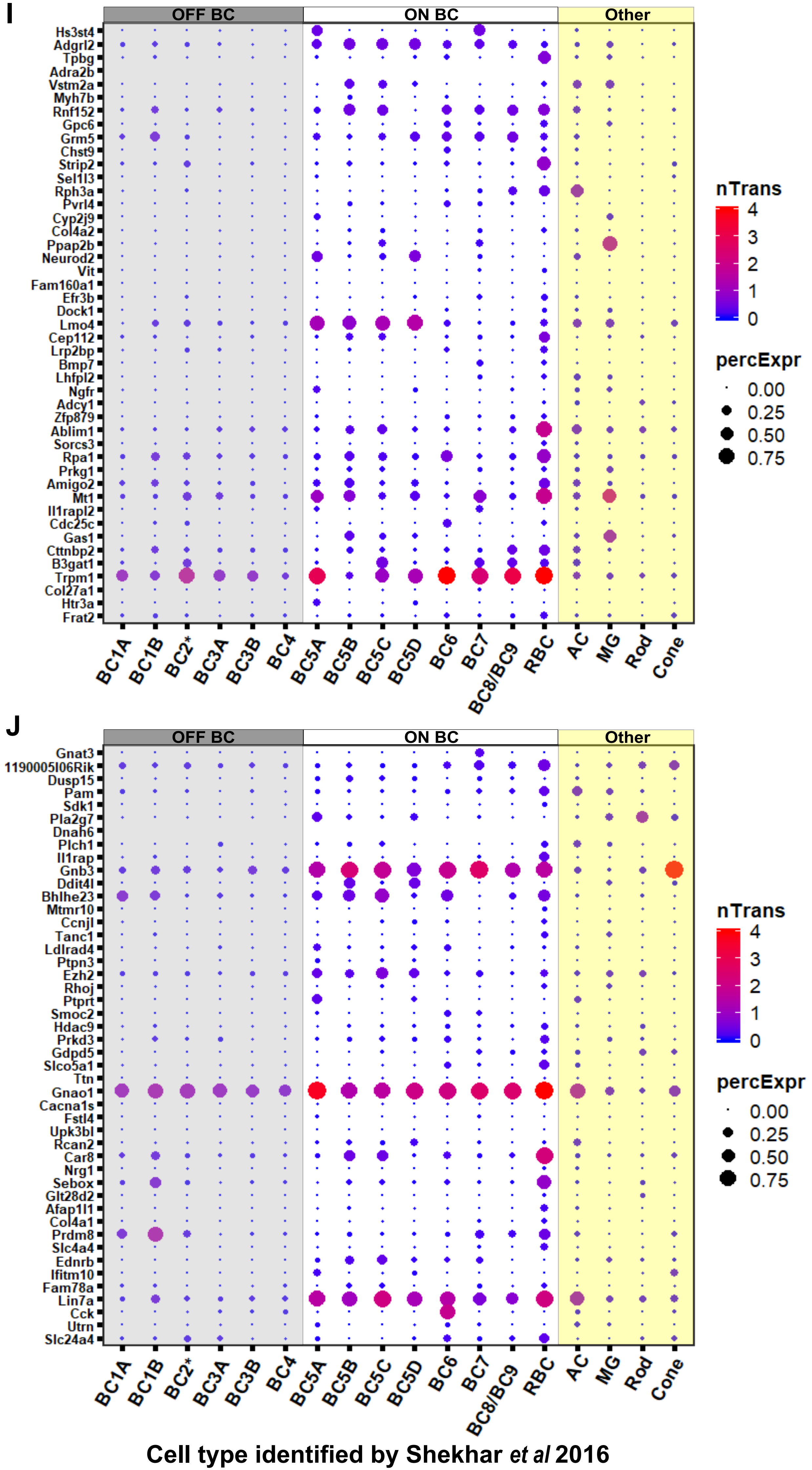
I
OFF BC
ON BC
Other
Hs3st4
Adgrl2
Tpbg
Adra2b
Vstm2a
Myh7b
Rnf152
Gpc6
Grm5
Chst9
Strip2
Sel1l3
Rph3a
Pvrl4
Cyp2j9
Col4a2
Ppap2b
Neurod2
Vit
Fam160a1
Efr3b
Dock1
Lmo4
Cep112
Lrp2bp
Bmp7
Lhfpl2
Ngfr
Adcy1
Zfp879
Ablim1
Sorcs3
Rpa1
Prkg1
Amigo2
Mt1
Il1rapl2
Cdc25c
Gas1
Cttnbp2
B3gat1
Trpm1
Col27a1
Htr3a
Frat2
BC1A
BC1B
BC2*
BC3A
BC3B
BC4
BC5A
BC5B
BC5C
BC5D
BC6
BC7
BC8/BC9
RBC
AC
MG
Rod
Cone
nTrans
4
3
2
1
0
percExpr
0.00
0.25
0.50
0.75
J
OFF BC
ON BC
Other
Gnat3
1190005I06Rik
Dusp15
Pam
Sdk1
Pla2g7
Dnah6
Plch1
Il1rap
Gnb3
Ddit4l
Bhlhe23
Mtmr10
Ccnjl
Tanc1
Ldlrad4
Ptpn3
Ezh2
Rhoj
Ptprt
Smoc2
Hdac9
Prkd3
Gdpd5
Slco5a1
Ttn
Gnao1
Cacna1s
Fstl4
Upk3bl
Rcan2
Car8
Nrg1
Sebox
Glt28d2
Afap1l1
Col4a1
Prdm8
Slc4a4
Ednrb
Ifitm10
Fam78a
Lin7a
Cck
Utrn
Slc24a4
BC1A
BC1B
BC2*
BC3A
BC3B
BC4
BC5A
BC5B
BC5C
BC5D
BC6
BC7
BC8/BC9
RBC
AC
MG
Rod
Cone
nTrans
4
3
2
1
0
percExpr
0.00
0.25
0.50
0.75
Cell type identified by Shekhar et al 2016

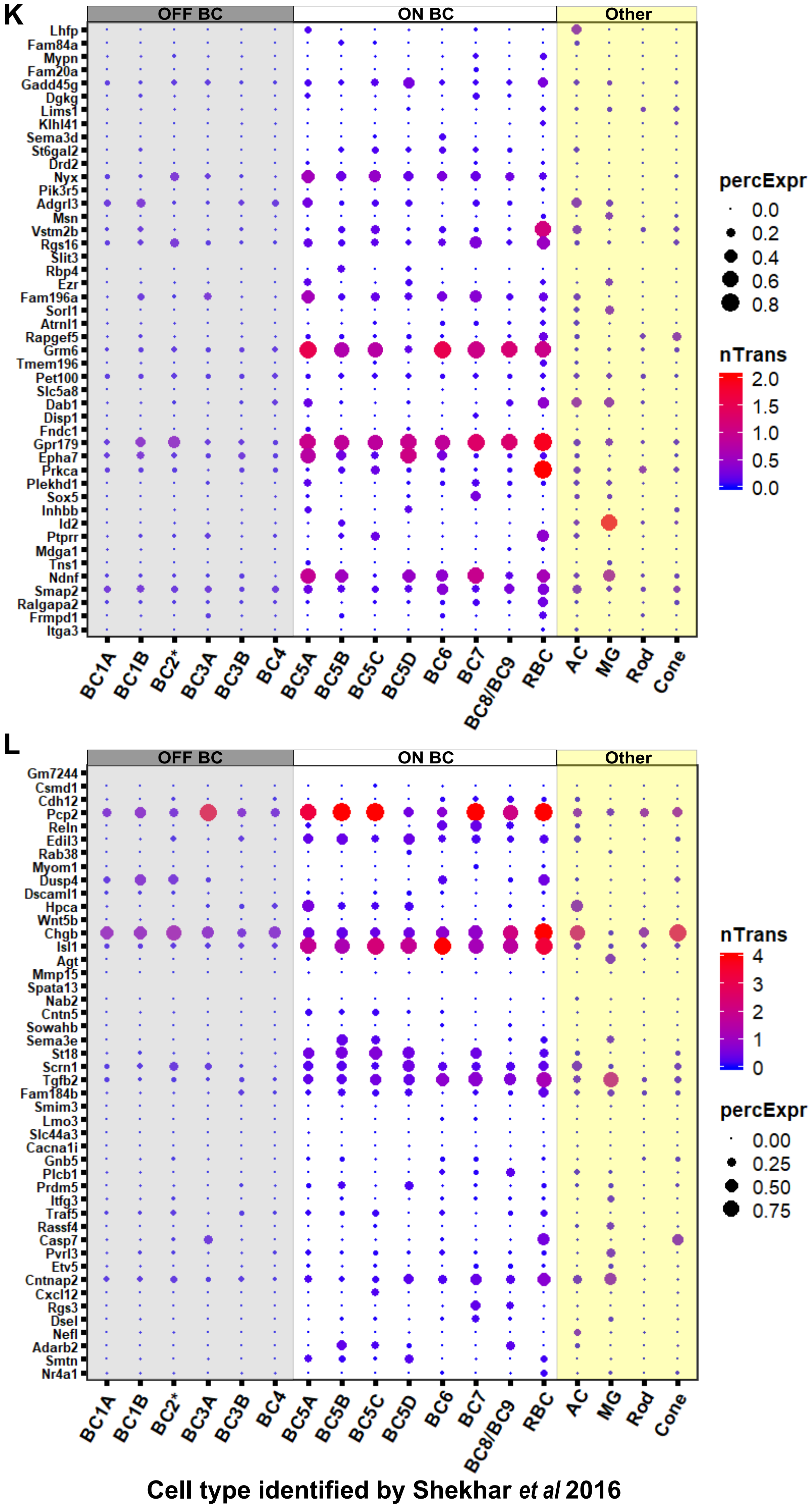
K
OFF BC
ON BC
Other
Lhfp
Fam84a
Mypn
Fam20a
Gadd45g
Dgkg
Lims1
Klhl41
Sema3d
St6gal2
Drd2
Nyx
Pik3r5
Adgrl3
Msn
Vstm2b
Rgs16
Slit3
Rbp4
Ezr
Fam196a
Sorl1
Atrnl1
Rapgef5
Grm6
Tmem196
Pet100
Slc5a8
Dab1
Disp1
Fndc1
Gpr179
Epha7
Prkca
Plekhd1
Sox5
Inhbb
Id2
Ptprr
Mdga1
Tns1
Ndnf
Smap2
Ralgapa2
Frmpd1
Itga3
BC1A
BC1B
BC2*
BC3A
BC3B
BC4
BC5A
BC5B
BC5C
BC5D
BC6
BC7
BC8/BC9
RBC
AC
MG
Rod
Cone
percExpr
0.0
0.2
0.4
0.6
0.8
nTrans
2.0
1.5
1.0
0.5
0.0
L
OFF BC
ON BC
Other
Gm7244
Csmd1
Cdh12
Pcp2
Reln
Edil3
Rab38
Myom1
Dusp4
Dscaml1
Hpca
Wnt5b
Chgb
Isl1
Agt
Mmp15
Spata13
Nab2
Cntn5
Sowahb
Sema3e
St18
Scrn1
Tgfb2
Fam184b
Smim3
Lmo3
Slc44a3
Cacna1i
Gnb5
Plcb1
Prdm5
Itfg3
Traf5
Rassf4
Casp7
Pvrl3
Etv5
Cntnap2
Cxcl12
Rgs3
Dsel
Nefl
Adarb2
Smtn
Nr4a1
BC1A
BC1B
BC2*
BC3A
BC3B
BC4
BC5A
BC5B
BC5C
BC5D
BC6
BC7
BC8/BC9
RBC
AC
MG
Rod
Cone
nTrans
4
3
2
1
0
percExpr
0.00
0.25
0.50
0.75
Cell type identified by Shekhar et al 2016

M

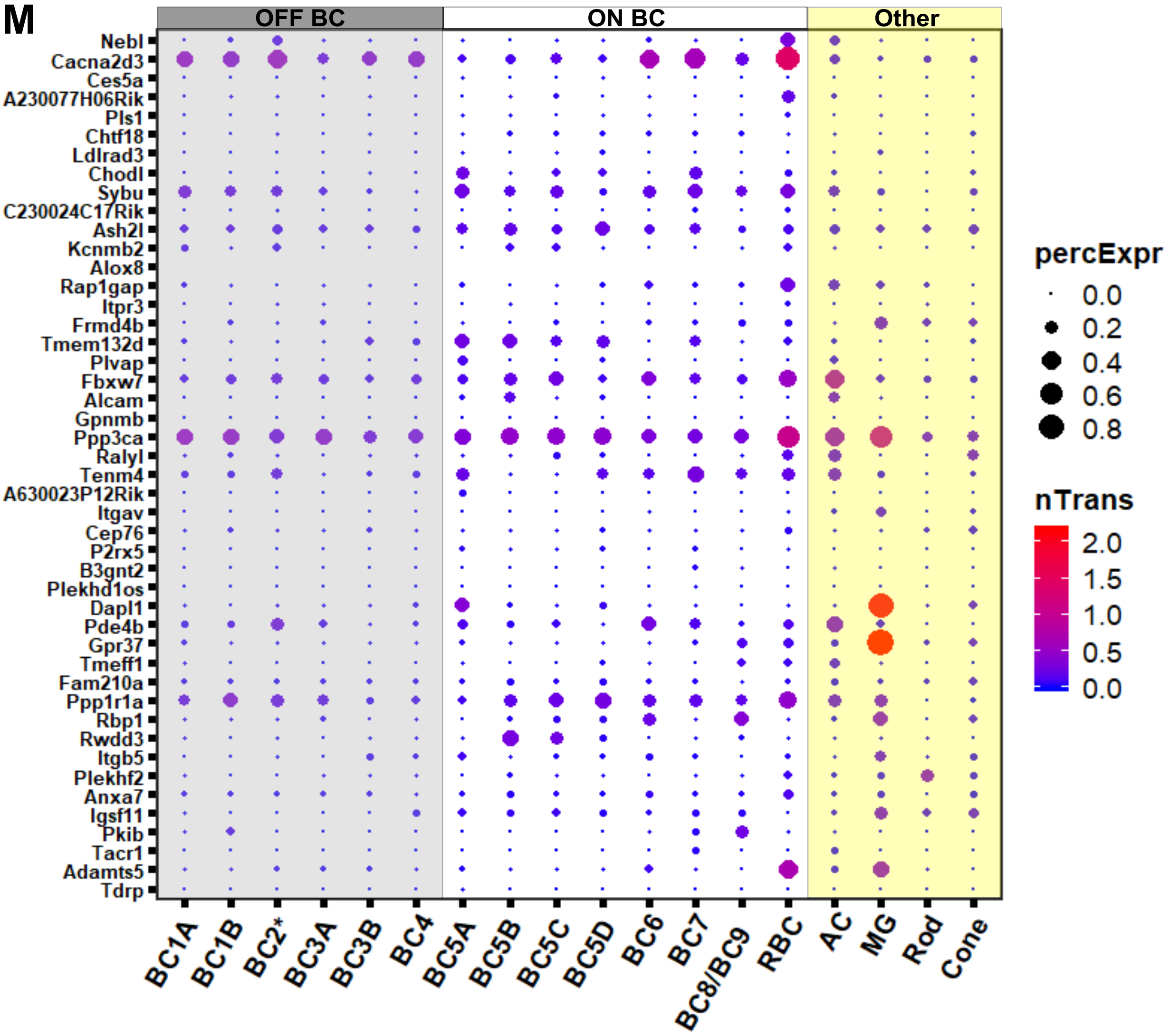

N

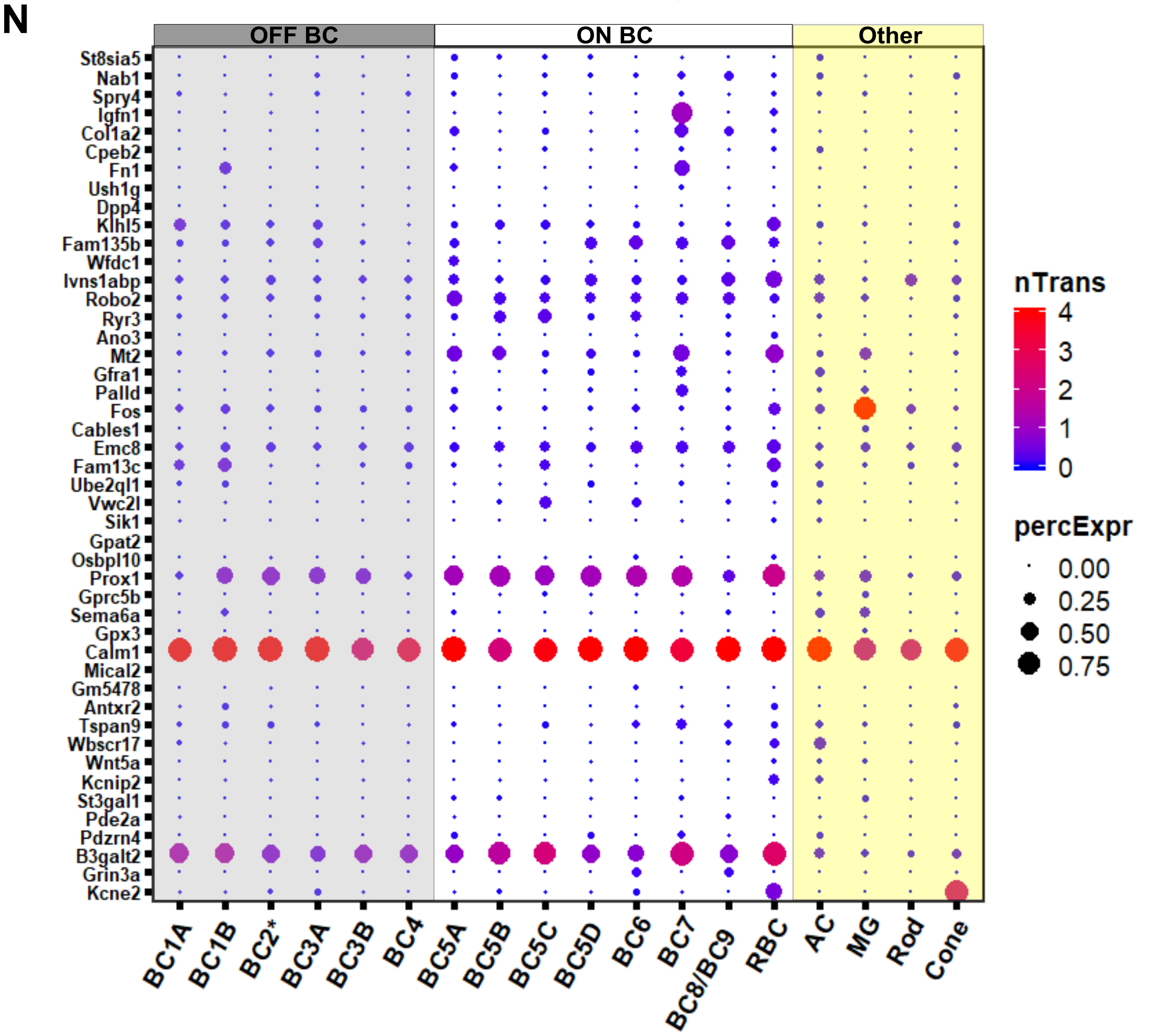

Cell type identified by Shekhar *et al* 2016

O

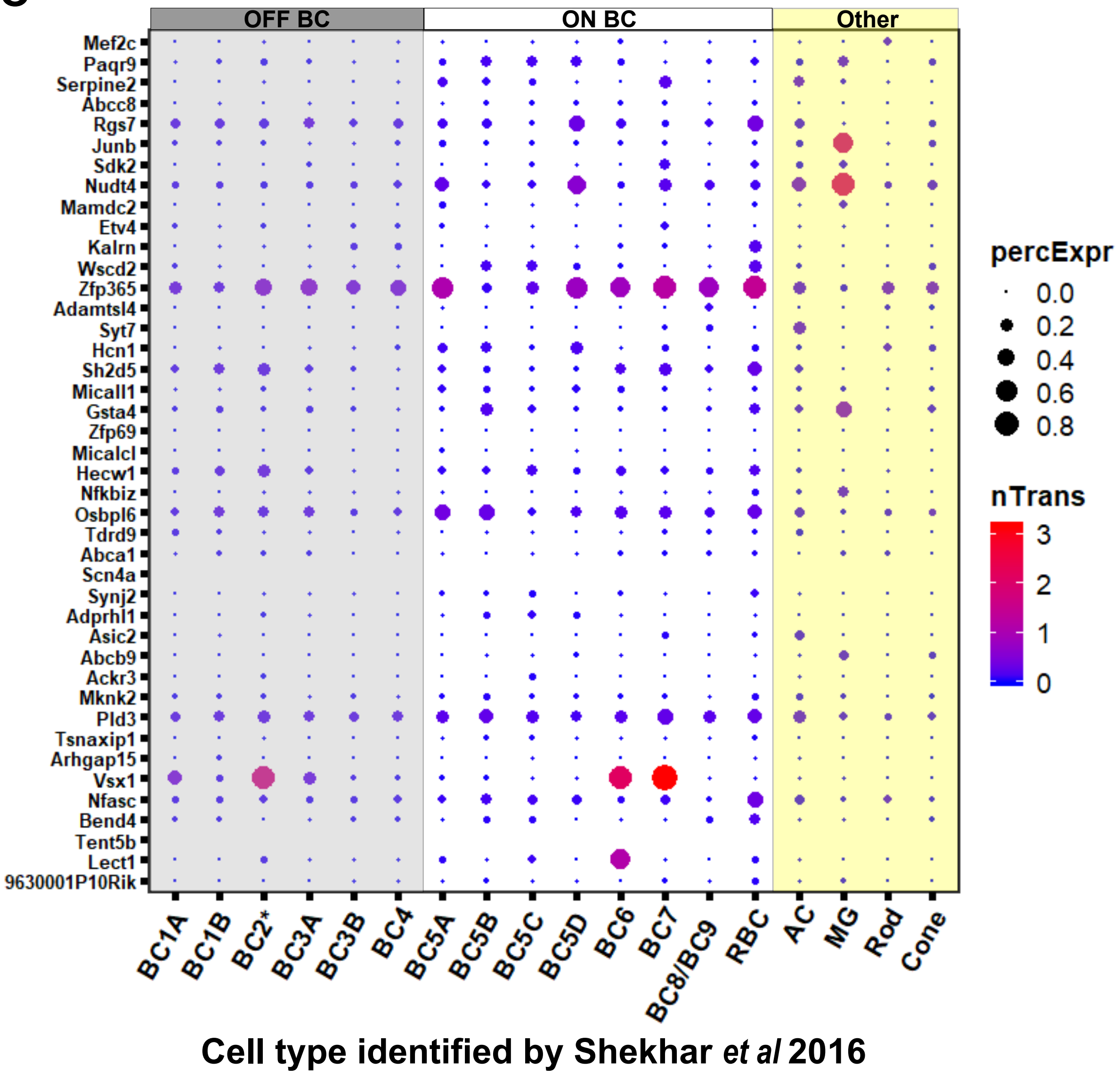
OFF BC
ON BC
Other
Mef2c
Paqr9
Serpine2
Abcc8
Rgs7
Junb
Sdk2
Nudt4
Mamdc2
Etv4
Kalrn
Wscd2
Zfp365
Adamtsl4
Syt7
Hcn1
Sh2d5
Micall1
Gsta4
Zfp69
Micalcl
Hecw1
Nfkbiz
Osbpl6
Tdrd9
Abca1
Scn4a
Synj2
Adprhl1
Asic2
Abcb9
Ackr3
Mknk2
Pld3
Tsnaxip1
Arhgap15
Vsx1
Nfasc
Bend4
Tent5b
Lect1
9630001P10Rik
BC1A
BC1B
BC2*
BC3A
BC3B
BC4
BC5A
BC5B
BC5C
BC5D
BC6
BC7
BC8/BC9
RBC
AC
MG
Rod
Cone
Cell type identified by Shekhar et al 2016
percExpr
0.0
0.2
0.4
0.6
0.8
nTrans
3
2
1
0
