# Supplementary figures and images for: Cis-regulatory basis of sister cell type divergence in the vertebrate retina
Source: eLife. 2019 Oct 21;8:e48216. doi: 10.7554/eLife.48216 (PMC6802965; doi:10.7554/eLife.48216)

**A**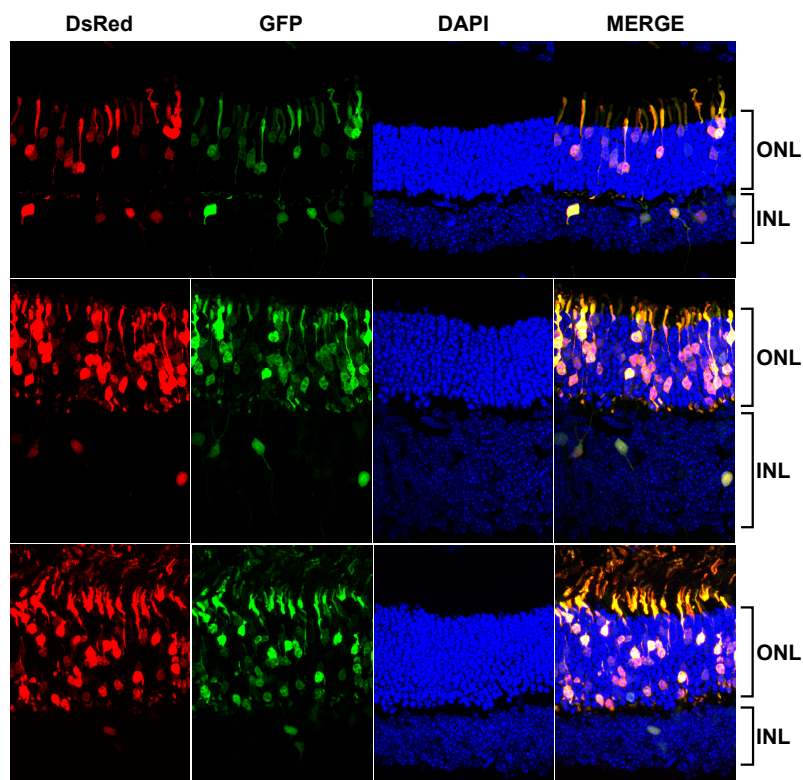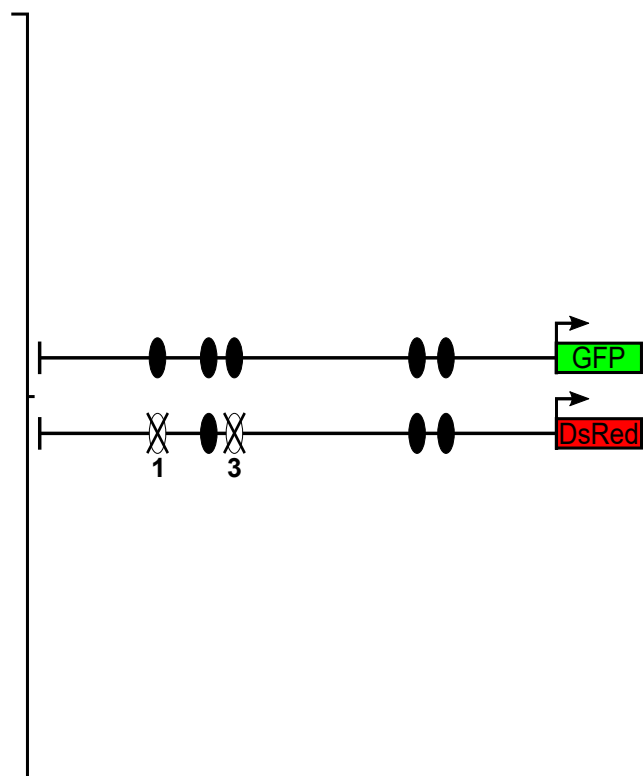**B**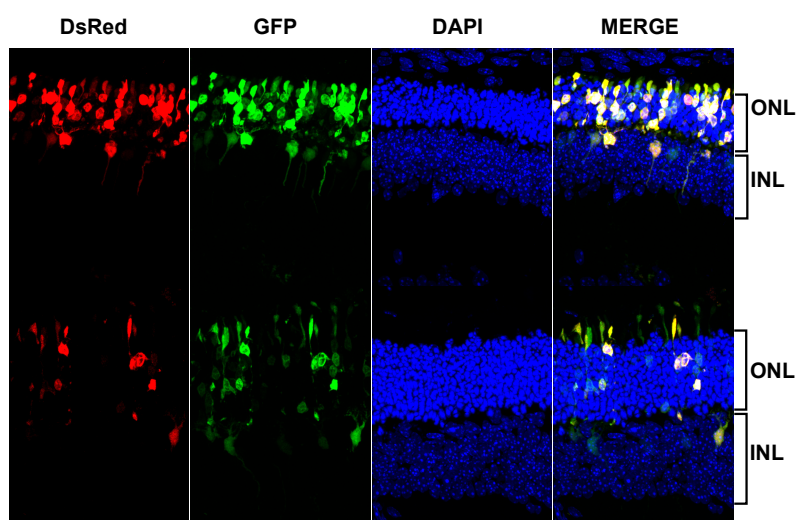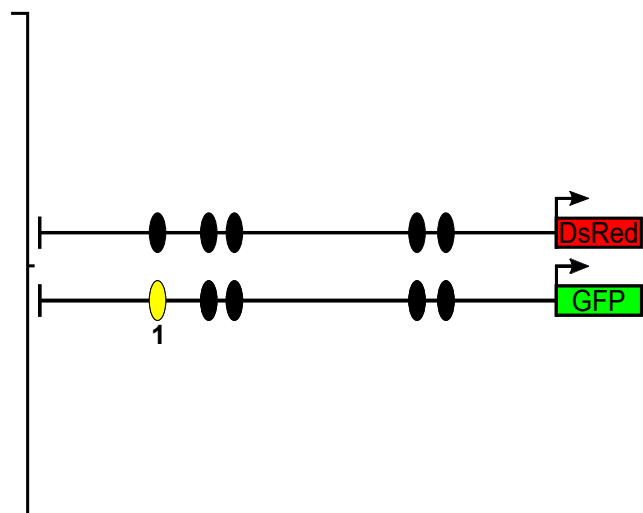**C**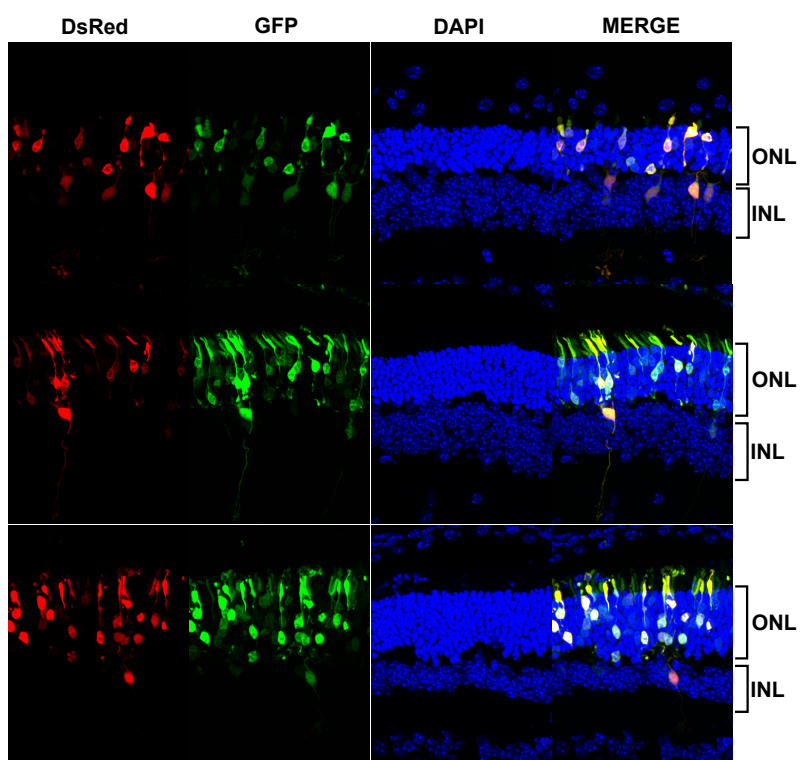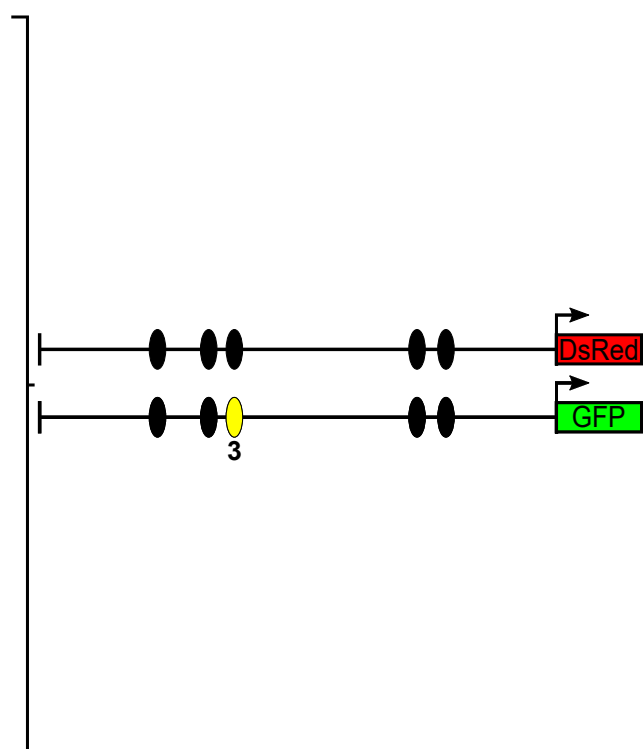

D

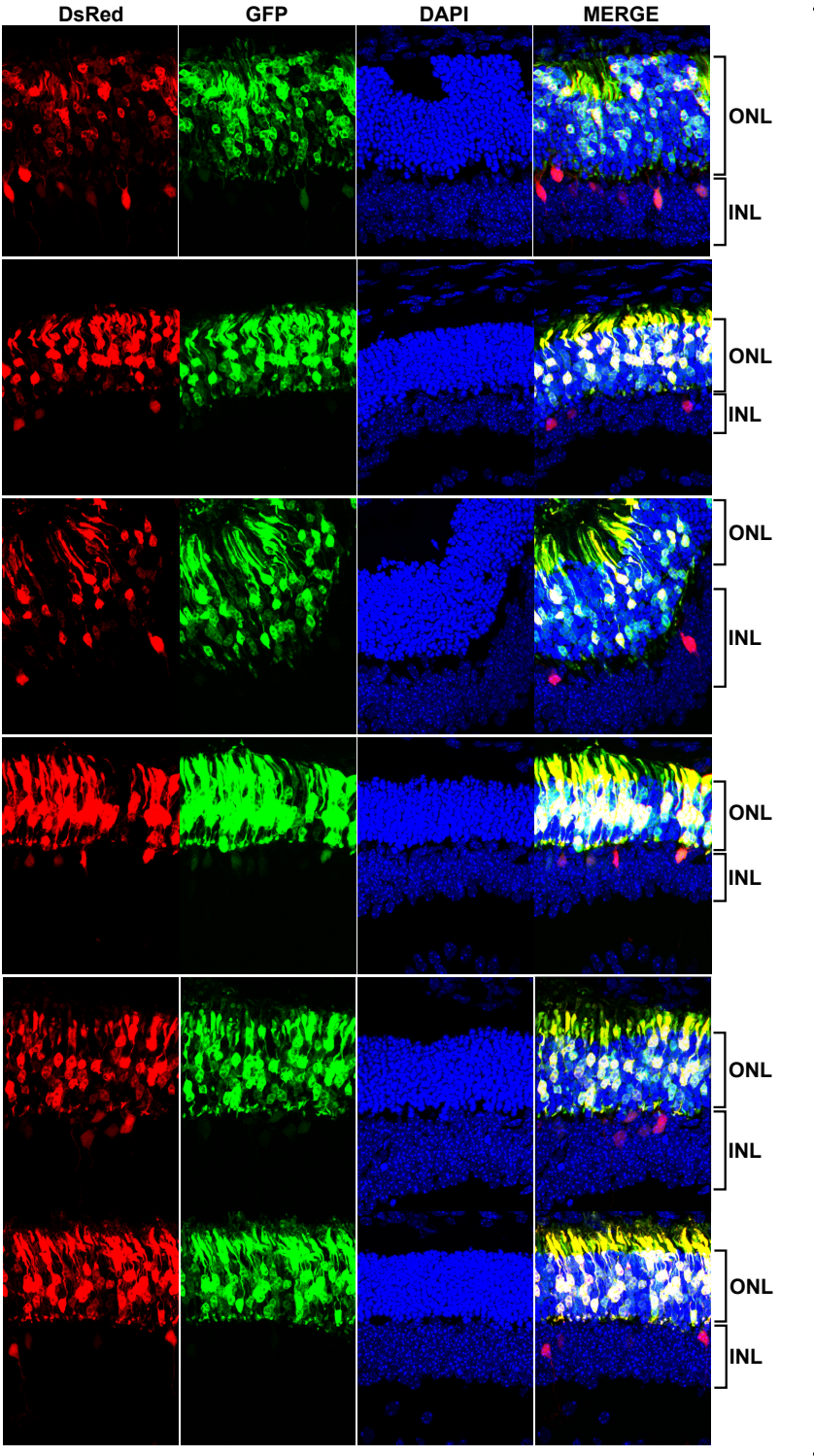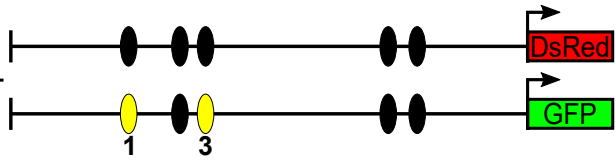

E

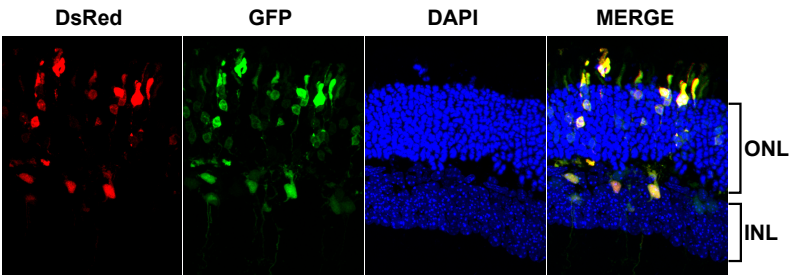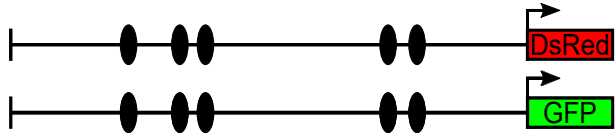

Supplement: Figure 6—source data 1. — Additional replicates from in vivo subretinal injection and electroporation of reporter pairs shown in Figure 6C. Schematics of each reporter pair are shown at the right of each panel, with WT (black), inactivated K50 (crossed out), and novel Q50 (yellow) motif sites shown as ellipses. [file elife-48216-fig6-data1.pdf]
