# Supplementary material for: Cis-regulatory basis of sister cell type divergence in the vertebrate retina
Source: eLife. 2019 Oct 21;8:e48216. doi: 10.7554/eLife.48216 (PMC6802965; doi:10.7554/eLife.48216)
Supplement: Transparent reporting form [file elife-48216-transrepform.docx]

***eLife’s* transparent reporting form**

We encourage authors to provide detailed information *within their submission* to facilitate the interpretation and replication of experiments. Authors can upload supporting documentation to indicate the use of appropriate reporting guidelines for health-related research (see [EQUATOR Network](http://www.equator-network.org/%20)), life science research (see the [BioSharing Information Resource](https://biosharing.org/)), or the [ARRIVE guidelines](http://www.plosbiology.org/article/info:doi/10.1371/journal.pbio.1000412) for reporting work involving animal research. Where applicable, authors should refer to any relevant reporting standards documents in this form.

If you have any questions, please consult our Journal Policies and/or contact us: [editorial@elifesciences.org](mailto:editorial@elifesciences.org).

**Sample-size estimation**

- You should state whether an appropriate sample size was computed when the study was being designed
- You should state the statistical method of sample size computation and any required assumptions
- If no explicit power analysis was used, you should describe how you decided what sample (replicate) size (number) to use

Please outline where this information can be found within the submission (e.g., sections or figure legends), or explain why this information doesn’t apply to your submission:

No power calculation was performed. The number of replicates for ATAC-seq and RNA-seq were based on those used in previous studies. For RNA-seq, technical analyses (Schurch, *et. al., RNA* 2016) demonstrate that three replicates is sufficient for identifying log_2_ fold-change differences >2 with DESeq2.

**Replicates**

- You should report how often each experiment was performed
- You should include a definition of biological versus technical replication
- The data obtained should be provided and sufficient information should be provided to indicate the number of independent biological and/or technical replicates
- If you encountered any outliers, you should describe how these were handled
- Criteria for exclusion/inclusion of data should be clearly stated
- High-throughput sequence data should be uploaded before submission, with a private link for reviewers provided (these are available from both GEO and ArrayExpress)

Please outline where this information can be found within the submission (e.g., sections or figure legends), or explain why this information doesn’t apply to your submission:

Information on replicates of ATAC-seq and RNA-seq is outlined in supplemental Table S1.

For each bipolar cell type, biological replicates derived from an independent FACS experiment.

Differentially expressed genes arising from low-level contamination of FAC-sorted bipolar cell populations with photoreceptor cells were filtered out as described in the Materials and Methods.

All raw ATAC-seq and RNA-seq data for bipolar cells is available through GEO accession GSE131625.

**Statistical reporting**

- Statistical analysis methods should be described and justified
- Raw data should be presented in figures whenever informative to do so (typically when N per group is less than 10)
- For each experiment, you should identify the statistical tests used, exact values of N, definitions of center, methods of multiple test correction, and dispersion and precision measures (e.g., mean, median, SD, SEM, confidence intervals; and, for the major substantive results, a measure of effect size (e.g., Pearson's r, Cohen's d)
- Report exact p-values wherever possible alongside the summary statistics and 95% confidence intervals. These should be reported for all key questions and not only when the p-value is less than 0.05.

Please outline where this information can be found within the submission (e.g., sections or figure legends), or explain why this information doesn’t apply to your submission:

Information on statistical reporting can be found throughout the results and materials and methods sections of the manuscript.

(For large datasets, or papers with a very large number of statistical tests, you may upload a single table file with tests, Ns, etc., with reference to sections in the manuscript.)

**Group allocation**

- Indicate how samples were allocated into experimental groups (in the case of clinical studies, please specify allocation to treatment method); if randomization was used, please also state if restricted randomization was applied
- Indicate if masking was used during group allocation, data collection and/or data analysis

Please outline where this information can be found within the submission (e.g., sections or figure legends), or explain why this information doesn’t apply to your submission:

N/A

**Additional data files (“source data”)**

- We encourage you to upload relevant additional data files, such as numerical data that are represented as a graph in a figure, or as a summary table
- Where provided, these should be in the most useful format, and they can be uploaded as “Source data” files linked to a main figure or table
- Include model definition files including the full list of parameters used
- Include code used for data analysis (e.g., R, MatLab)
- Avoid stating that data files are “available upon request”

Please indicate the figures or tables for which source data files have been provided:

Supplementary Data for ATAC-seq is included in tables S4, S7 and S8. The annotated set of retinal ATAC-seq peaks used to create Figure 3B is included in table S4, along with lists of peaks identified in each bipolar cell ATAC-seq replicate. Table S7 includes annotated lists of differentially accessible ATAC-seq peaks which are shown in Figure 5 and Figure S5. Table S8 includes known motif enrichment results for each bipolar cell population, as well as a comparison between enrichment in pan-BC and photoreceptor populations for each motif, which were used to identify those motifs shown in Figure 4, Figure S4, and Figure 5D.

Differential expression data, including pan-BC versus photoreceptor and ON-BC versus OFF-BC is included in table S5. This includes the list of genes shown in Figure 2 and Figure S2.

Figures 2, 3A, 3B, 4 and 5 were created in R and are described in the Materials and Methods.
